# Association between 12 composite inflammatory indices and postoperative complications in colorectal cancer surgery: a two-center retrospective study

**DOI:** 10.3389/fonc.2026.1808454

**Published:** 2026-04-22

**Authors:** Chunpeng Pan, Jiwei Yu, Shuai Huang, Shoulian Wang, Xiaochun Ni, Haibo Wang, Chihao Zhang, Fan Xiao

**Affiliations:** 1 Department of General Surgery, Shanghai Ninth People’s Hospital, School of Medicine, Shanghai Jiao Tong University, Shanghai, China; 2 Department of Hepatic Surgery, Eastern Hepatobiliary Surgery Hospital, Navy Military Medical University, Shanghai, China

**Keywords:** colorectal cancer, composite inflammatory indices, postoperative complications, receiver operating characteristic, retrospective study

## Abstract

**Background:**

Postoperative complications (POCs) remain a major challenge in colorectal cancer (CRC) surgery. Easily accessible composite inflammatory indices derived from routine blood tests hold promise for preoperative risk stratification, but their comparative predictive value for POCs is unclear.

**Methods:**

We conducted a two-center, retrospective study of 1331 patients who underwent radical CRC surgery between January 2021 to October 2025. The associations between preoperative composite inflammatory indices and the complications after surgery were evaluated using logistic regression. Their predictive discriminative performances were further assessed and compared using receiver operating characteristic (ROC) curve analysis, with the areas under the curve (AUC) compared by DeLong’s test.

**Results:**

The complication rate was 17.2% (229/1331). Patients with complications exhibited a significantly more pronounced pro-inflammatory and immunosuppressive preoperative profile across all indices (all *P* < 0.05). After adjustment, the systemic immune-inflammation index (SII), neutrophil-to-lymphocyte ratio (NLR), C-reactive protein-to-lymphocyte ratio (CLR), and systemic inflammation response index (SIRI) were identified as the strongest independent predictors, with the highest quartile (Q4) conferring substantially increased risks (OR for SII: 7.51; for NLR: 5.86; for CLR: 2.24; for SIRI: 2.99; all *P*-trend <0.05). ROC analysis confirmed SII as the best single predictor (AUC: 0.738). A combined model integrating SII, NLR, CLR, and SIRI achieved superior discriminative ability (AUC: 0.792), significantly outperforming any single index (all *P* < 0.05).

**Conclusion:**

The SII, NLR, CLR and SIRI, are strongly associated with the risk of POCs after CRC surgery. A model combining these four indices provides superior predictive performance.

## 1 Introduction

Colorectal cancer (CRC) represents a major global health burden, being the third most commonly diagnosed malignancy and the second leading cause of cancer-related mortality worldwide (1). For patients with localized or locally advanced disease, surgical resection remains the cornerstone of curative-intent treatment, with continuous refinements in surgical techniques and perioperative management (2). Despite these advances, postoperative complications (POCs) continue to pose a significant clinical challenge, affecting 15-20% of patients undergoing major colorectal resection (3). These complications, which include infectious events such as surgical site infection and anastomotic leakage, cardiopulmonary incidents, and thromboembolic events, are critical determinants of both short-term recovery and long-term prognosis (4–6). They are strongly associated with prolonged hospital stays, increased healthcare costs, reduced quality of life, higher readmission rates, and may adversely affect oncological survival by delaying adjuvant therapy and fostering a pro-metastatic environment (7, 8).

The pathophysiology of POCs is complex and multifactorial, involving patient-specific factors, surgical trauma, and dynamic biological responses (9). Among these, the systemic inflammatory response (SIR) is a key and potentially modifiable component. An excessive or dysregulated postoperative inflammatory response may impair tissue healing, increase infection risk, and contribute to organ dysfunction (10). Therefore, a patient’s inflammatory status, both before and immediately after surgery, is a crucial biological determinant of postoperative resilience and recovery. Traditionally, the assessment of systemic inflammation has relied on single hematological and biochemical parameters, such as neutrophil count, lymphocyte count, platelet count, C-reactive protein (CRP), and albumin levels (11, 12).

However, these individual markers provide only a partial view of the complex immune-nutritional landscape. Recognizing this limitation, there has been a paradigm shift toward integrated biomarkers that combine multiple pathways into single, potentially more robust prognostic indices. Substantial evidence has linked several composite inflammatory indices, particularly the neutrophil-to-lymphocyte ratio (NLR), platelet-to-lymphocyte ratio (PLR), systemic immune-inflammation index (SII), and C-reactive protein-to-albumin ratio (CAR), to long-term oncological outcomes in CRC (13–15). Moreover, the predictive utility of newer indices, such as the pan-immune inflammation value (PIV), hemoglobin-albumin-lymphocyte-platelet (HALP) score, C-reactive protein-albumin-lymphocyte (CALLY) index, and neutrophil-to-platelet ratio (NPR), in this specific clinical context remains largely unexplored. Addressing this gap is clinically imperative.

Therefore, the primary aim of this two-center retrospective study was to systematically evaluate 12 distinct composite inflammatory indices: SII, NLR, PLR, LMR, NPR, SIRI, PAR, CAR, CLR, CALLY, PIV, and HALP. We sought to determine which index or combination of indices is most strongly associated with clinically relevant POCs after curative-intent CRC surgery. By leveraging a dual-institutional dataset, this study seeks to determine the comparative prognostic value of these indices, validate emerging biomarkers, and identify the most potent hematological indicator of postoperative vulnerability.

## 2 Methods

### 2.1 Study design and patients

This two-center retrospective study utilized data from CRC surgery databases and electronic medical records at Shanghai Ninth People’s Hospital and Eastern Hepatobiliary Surgery Hospital. The study included patients who underwent laparoscopic or robotic-assisted surgical resection for CRC between January 2021 and October 2025.

Inclusion criteria (1): Age ≥ 18 years (2); Underwent elective, curative-intent laparoscopic or robotic-assisted radical resection for primary colorectal adenocarcinoma (3); Had histopathological confirmation of colorectal adenocarcinoma (4); Underwent surgery between January 1, 2020, and October 31, 2025 (5); Possessed complete preoperative laboratory data within 7 days before surgery. Exclusion criteria (1): Emergency surgery, palliative resection, or local excision only; (2) Previous neoadjuvant chemotherapy or radiotherapy; (3) Concurrent conditions that could substantially confound inflammatory markers, including active infection, inflammatory bowel disease, other active malignancies, or known hematological disorders; (4) Missing essential perioperative data or 30-day postoperative follow-up information on complications.

**Figure 1:**
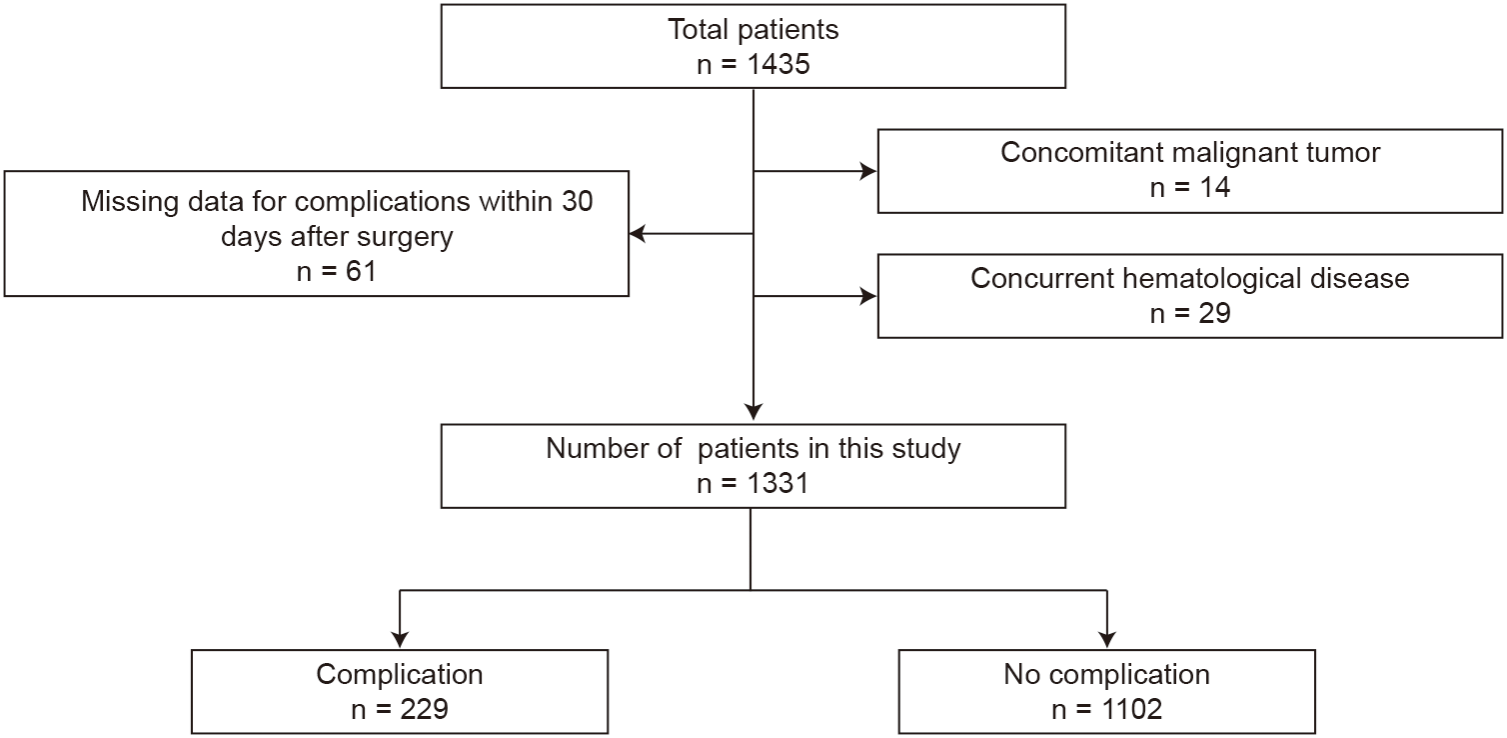
The flowchart of the study population.

The detailed patient screening process is summarized in **Figure 1**. Finally, a total of 1331 patients were included in this study. This study was approved by the Ethics Committee of the Shanghai Ninth People’s Hospital (No. No. SH9H-2025-T525-1) and each participating institution.

### 2.2 Data collection

Data were systematically extracted from the electronic medical records and prospectively maintained surgical databases of all participating centers using a standardized case report form. Collected variables encompassed patient demographics and baseline characteristics, including age, gender, body mass indices (BMI), smoking history, and alcohol consumption. Preoperative comorbidities of interest comprised a documented history of diabetes mellitus, hypertension, and cardiovascular disease. Primary tumor location, operative approach, and total operative time, were recorded. Crucially, all laboratory parameters used for calculating the inflammatory indices were obtained from routine preoperative blood tests performed within the 7 days preceding surgery. Furthermore, comprehensive details on any POC occurring within the 30-day postoperative period were meticulously collected to enable accurate outcome assessment and grading. To ensure data quality and consistency, the extraction process was performed independently by two researchers, with any discrepancies resolved through consensus discussion or by adjudication from a senior investigator.

### 2.3 Calculation of composite inflammatory indices

All composite inflammatory indices were calculated using routine preoperative peripheral blood test results, extracted from the laboratory database. The following hematological and biochemical parameters, obtained within 7 days before surgery, were utilized: neutrophil count (×10^9^/L), lymphocyte count (×10^9^/L), monocyte count (×10^9^/L), platelet count (×10^9^/L), hemoglobin level (g/L), albumin level (g/L), and CRP level (mg/L). The twelve indices were computed according to their established formulas (16):

SII: Platelet count × Neutrophil count/Lymphocyte count.
NLR: Neutrophil count/Lymphocyte count.
PLR: Platelet count/Lymphocyte count.
LMR: Lymphocyte count/Monocyte count.
NPR: Neutrophil count/Platelet count.
SIRI: Neutrophil count × Monocyte count/Lymphocyte count.
PIV: Neutrophil count × Monocyte count × Platelet count/Lymphocyte count.
PAR: Platelet count/Albumin level.
CAR: CRP level/Albumin level.
CLR: CRP level/Lymphocyte count.
CALLY: Albumin level × Lymphocyte count/(CRP level × 10).
HALP: Hemoglobin level × Albumin level × Lymphocyte count/Platelet count.

### 2.4 Outcome measurement

The outcome measurement of this study was the occurrence of any POC within 30 days following CRC surgery. The severity of each recorded complication was graded using the modified Clavien-Dindo classification (CDC) (17). In this study, a clinically significant complication was defined as a CDC grade ≥II, representing events that necessitate pharmacological, endoscopic, or surgical intervention beyond routine postoperative care (18).

### 2.5 Statistical analysis

Continuous variables are expressed as mean ± standard deviation (SD). Normality was assessed using the Shapiro-Wilk test. Differences between groups were analyzed with the independent samples t-test for normally distributed data and the Mann-Whitney U test for non-normally distributed data. Categorical variables are presented as frequencies and percentages, and group differences were evaluated using the chi-square test or Fisher’s exact test, as appropriate.

Multicollinearity among covariates was assessed using the variance inflation factor (VIF), with a threshold of <5 indicating no significant multicollinearity. Each composite inflammatory index was entered as an independent variable in separate binary logistic regression models, categorized by quartiles (Q1: ≤25th percentile; Q2: >25th to ≤50th percentile; Q3: >50th to ≤75th percentile; Q4: >75th percentile). Two models were constructed for each index: Model 1 was unadjusted, and Model 2 was adjusted for covariates, with no mutual adjustment among the inflammatory indices. Results are reported as odds ratios (ORs) with 95% confidence intervals (CIs). Receiver operating characteristic (ROC) curve analysis was performed using the continuous measured values of each inflammatory index to assess discriminative performance, with the area under the curve (AUC) calculated and compared using the DeLong test. Optimal cut-off values were determined based on the Youden index (sensitivity + specificity – 1).

All analyses were performed using R 4.3.3 and SPSS 26.0. A two-sided *P*-value <0.05 was considered statistically significant.

## 3 Results

### 3.1 Patient characteristics

A total of 1331 patients who underwent radical resection for CRC were included in the final analysis. According to the presence of 30-day POCs, the cohort was stratified into two groups: the complication group (n=229, 17.2%) and the no complication group (n=1102, 82.8%). A detailed breakdown of the types and severity of POCs is presented in **Table 1**.

**Table 1:** Types and severity of POCs (n=229).

Type	n (%)	CDC grade		
		II	III	IV
Surgical site infection	68 (29.7)	45 (19.7)	23 (10.0)	0 (0.0)
Anastomotic leakage	32 (14.0)	0 (0.0)	27 (11.8)	5 (2.2)
Intra-abdominal abscess	24 (10.5)	15 (6.6)	9 (3.9)	0 (0.0)
Cardiopulmonary complications	51 (22.3)	35 (15.3)	12 (5.2)	4 (1.7)
Thromboembolic events	18 (7.9)	12 (5.2)	6 (2.6)	0 (0.0)
Others	36 (15.7)	28 (12.2)	7 (3.1)	1 (0.4)

CDC, Clavien-Dindo classification.

The baseline demographic and clinicopathological characteristics of the two groups are summarized in **Table 2**. Patients who developed POCs were significantly older, had a higher BMI, and underwent longer operative procedures compared to those without complications (all *P* < 0.05). Furthermore, a significantly higher prevalence of modifiable risk factors, including smoking and alcohol use, as well as comorbidities such as diabetes and cardiovascular disease, was observed in the complication group (all *P* < 0.05). No significant intergroup differences were found in terms of gender, hypertension, tumor location, or surgical approach (all *P >*0.05).

**Table 2:** Comparison of characteristics between complication and no complication group.

Variables	Complication (n= 229)	No complication (n= 1102)	*t*/*χ^2^*	*P*
Age (years)	65.9 ± 8.7	64.3 ± 10.2	-2.133	0.033
BMI (kg/m^2^)	24.6 ± 3.6	23.5 ± 3.6	-4.280	<0.001
Operation time (min)	178.8 ± 60.4	163.1 ± 47.5	-4.326	<0.001
Gender			0.184	0.668
Male	133 (58.1)	623 (56.5)		
Female	96 (41.9)	479 (43.5)		
Smoking	73 (31.9)	276 (25.0)	4.575	0.032
Alcohol use	65 (28.4)	244 (22.1)	4.145	0.042
Diabetes	42 (18.3)	132 (12.0)	6.754	0.009
Hypertension	90 (39.3)	419 (38.0)	0.131	0.717
Cardiovascular disease	34 (14.8)	87 (7.9)	11.089	0.001
Tumor location			0.270	0.603
Colon	121 (52.8)	603 (54.7)		
Rectum	108 (47.2)	499 (45.3)		
Operative approach			2.004	0.157
Laparoscopy	194 (84.7)	971 (88.1)		
Robot	35 (15.3)	131 (11.9)		

BMI, Body mass indices.

Preoperative peripheral blood indices and composite inflammatory indices are presented in **Table 3**. Strikingly, all evaluated parameters demonstrated highly significant differences between the two groups (all *P* < 0.05). This was characterized by significantly elevated levels of neutrophils, monocytes, platelets, CRP, SII, NLR, PLR, NPR, SIRI, PIV, PAR, CAR, and CLR in the complication group. Conversely, levels of lymphocytes, hemoglobin, albumin, LMR, CALLY, and HALP were significantly lower in the complication group.

**Table 3:** Comparison of preoperative peripheral blood indices and composite inflammatory indices between complication and no complication group.

Variables	Complication (n= 229)	No complication (n= 1102)	*t*/*χ^2^*	*P*
Peripheral blood indices				
Neutrophil (×10^9^/L)	7.21 ± 2.82	4.64 ± 1.75	17.916	<0.001
Lymphocyte (×10^9^/L)	1.31 ± 0.52	1.85 ± 0.68	-11.346	<0.001
Monocyte (×10^9^/L)	0.61 ± 0.34	0.55 ± 0.27	2.917	0.004
Platelet (×10^9^/L)	274.11 ± 80.25	247.37 ± 75.63	4.817	<0.001
Hemoglobin (g/L)	118.75 ± 18.22	127.46 ± 15.34	-7.557	<0.001
Albumin (g/L)	37.55 ± 4.86	41.85 ± 3.94	-14.397	<0.001
CRP (mg/L)	35.12 ± 18.26	8.51 ± 10.15	30.689	<0.001
Composite inflammatory indices				
SII	1208.99 ± 384.32	592.52 ± 189.45	36.171	<0.001
NLR	6.24 ± 3.51	2.83 ± 1.65	22.464	<0.001
PLR	225.02 ± 110.12	145.12 ± 70.05	14.034	<0.001
LMR	2.31 ± 1.24	3.53 ± 1.55	-11.189	<0.001
NPR	0.03 ± 0.01	0.02 ± 0.01	13.770	<0.001
SIRI	3.36 ± 2.52	1.38 ± 0.89	20.635	<0.001
PIV	921.45 ± 282.11	340.21 ± 148.34	44.822	<0.001
PAR	7.44 ± 2.84	5.93 ± 2.01	9.559	<0.001
CAR	1.01 ± 0.52	0.25 ± 0.12	43.334	<0.001
CLR	30.06 ± 11.04	5.54 ± 3.01	63.338	<0.001
CALLY	4.21 ± 2.07	15.13 ± 7.56	-21.684	<0.001
HALP	321.03 ± 155.12	481.30 ± 182.01	-12.420	<0.001

CRP, C-reactive protein; SII, Systemic immune-inflammation index; NLR, Neutrophil-to-lymphocyte ratio; PLR, Platelet-to-lymphocyte ratio; LMR, Lymphocyte-to-monocyte ratio; NPR, Neutrophil-to-platelet ratio; SIRI, Systemic inflammation response index; PIV, Pan-immune inflammation value; PAR, Platelet-to-albumin ratio; CAR, C-reactive protein-to-albumin ratio; CLR, C-reactive protein to lymphocyte ratio; CALLY, C-reactive protein-albumin-lymphocyte indices; HALP, Hemoglobin-albumin-lymphocyte-platelet.

### 3.2 Association of preoperative inflammatory indices with POCs

Multicollinearity diagnostics revealed no significant collinearity among covariates, with all VIF values < 2.0. The associations between the quartile levels of preoperative inflammatory indices and the risk of POCs are detailed in **Table 4**. After adjustment for age, BMI, operation time, smoking, alcohol use, diabetes, and cardiovascular disease in Model 2, the SII, NLR, PLR, SIRI, and CLR remained strong and independent predictors. Patients in the highest quartile (Q4) of these indices faced a substantially elevated risk, with OR of 7.51 for SII, 5.86 for NLR, 3.14 for PLR, 2.99 for SIRI, and 2.24 for CLR (all *P* for trend <0.05). Conversely, a higher LMR was associated with a significantly reduced risk (OR for Q4: 0.45; *P* for trend <0.05).

**Table 4:** Results of the logistic regression.

Item	OR (95% CI)				*P* for trend
	Q1	Q2	Q3	Q4	
SII					
Model 1	1.00 (Reference)	2.14 (1.22, 3.74)	3.80 (2.24, 6.43)	7.14 (4.30, 11.87)	<0.001
Model 2	1.00 (Reference)	2.26 (1.27, 3.99)	3.88 (2.27, 6.65)	7.51 (4.46, 12.64)	<0.001
NLR					
Model 1	1.00 (Reference)	1.77 (1.11, 2.83)	2.40 (1.52, 3.79)	4.36 (2.82, 6.74)	<0.001
Model 2	1.00 (Reference)	2.22 (1.36, 3.63)	3.15 (1.94, 5.11)	5.86 (3.66, 9.37)	<0.001
PLR					
Model 1	1.00 (Reference)	1.38 (0.88, 2.15)	1.81 (1.12, 2.78)	2.78 (1.84, 4.20)	<0.001
Model 2	1.00 (Reference)	1.49 (0.94, 2.36)	1.92 (1.23, 3.00)	3.14 (2.04, 4.82)	<0.001
LMR					
Model 1	1.00 (Reference)	0.89 (0.61, 1.30)	0.55 (0.37, 0.83)	0.46 (0.31, 0.70)	<0.001
Model 2	1.00 (Reference)	0.84 (0.56, 1.24)	0.51 (0.33, 0.77)	0.45 (0.30, 0.69)	<0.001
NPR					
Model 1	1.00 (Reference)	0.92 (0.62, 1.39)	0.92 (0.62, 1.38)	1.01 (0.71, 1.58)	0.812
Model 2	1.00 (Reference)	0.95 (0.63, 1.45)	0.98 (0.65, 1.48)	1.09 (0.72, 1.66)	0.735
SIRI					
Model 1	1.00 (Reference)	1.32 (0.84, 2.10)	1.98 (1.27, 3.09)	2.90 (1.89, 4.44)	<0.001
Model 2	1.00 (Reference)	1.31 (0.84, 2.05)	1.84 (1.19, 2.83)	2.99 (1.97, 4.53)	<0.001
PIV					
Model 1	1.00 (Reference)	1.25 (0.82, 1.91)	1.49 (0.99, 2.25)	1.53 (1.02, 2.31)	0.042
Model 2	1.00 (Reference)	1.20 (0.78, 1.86)	1.46 (0.96, 2.23)	1.58 (1.04, 2.42)	0.035
PAR					
Model 1	1.00 (Reference)	1.05 (0.69, 1.59)	1.42 (0.94, 2.13)	1.38 (0.91, 2.08)	0.058
Model 2	1.00 (Reference)	1.07 (0.69, 1.65)	1.45 (0.95, 2.21)	1.47 (0.96, 2.25)	0.048
CAR					
Model 1	1.00 (Reference)	1.04 (0.68, 1.57)	1.13 (0.75, 1.69)	1.74 (1.17, 2.58)	0.002
Model 2	1.00 (Reference)	0.99 (0.65, 1.52)	1.16 (0.77, 1.77)	1.63 (1.08, 2.45)	0.005
CLR					
Model 1	1.00 (Reference)	1.26 (0.80, 1.99)	2.15 (1.39, 3.32)	2.26 (1.47, 3.48)	<0.001
Model 2	1.00 (Reference)	1.24 (0.80, 1.93)	2.07 (1.36, 3.15)	2.24 (1.48, 3.40)	<0.001
CALLY					
Model 1	1.00 (Reference)	1.17 (0.78, 1.74)	0.84 (0.56, 1.27)	0.77 (0.51, 1.16)	0.245
Model 2	1.00 (Reference)	1.25 (0.82, 1.89)	0.91 (0.59, 1.39)	0.87 (0.57, 1.34)	0.412
HALP					
Model 1	1.00 (Reference)	0.87 (0.59, 1.30)	0.67 (0.45, 0.99)	0.62 (0.41, 0.93)	0.010
Model 2	1.00 (Reference)	0.81 (0.53, 1.22)	0.62 (0.41, 0.94)	0.60 (0.40, 0.91)	0.005

SII, Systemic immune-inflammation index; NLR, Neutrophil-to-lymphocyte ratio; PLR, Platelet-to-lymphocyte ratio; LMR, Lymphocyte-to-monocyte ratio; NPR, Neutrophil-to-platelet ratio; SIRI, Systemic inflammation response index; PIV, Pan-immune inflammation value; PAR, Platelet-to-albumin ratio; CAR, C-reactive protein-to-albumin ratio; CLR, C-reactive protein-to-lymphocyte ratio; CALLY, C-reactive protein-albumin-lymphocyte indices; HALP, Hemoglobin-albumin-lymphocyte-platelet; Model 1, Unadjusted covariates; Model 2, Adjusted for age, BMI, operation time, smoking, alcohol use, diabetes, and cardiovascular disease.

The CAR also showed a significant association, although its risk increase was most pronounced in the highest quartile (OR for Q4: 1.63; *P* for trend <0.05). In contrast, no significant linear trends were observed for the NPR, PIV, PAR, or the CALLY after multivariable adjustment (all *P* for trend >0.05). The HALP score exhibited a significant protective trend (OR for Q4: 0.60; *P* for trend <0.05).

### 3.3 Predictive performance and comparative analysis of preoperative inflammatory indices

**Figure 2:**
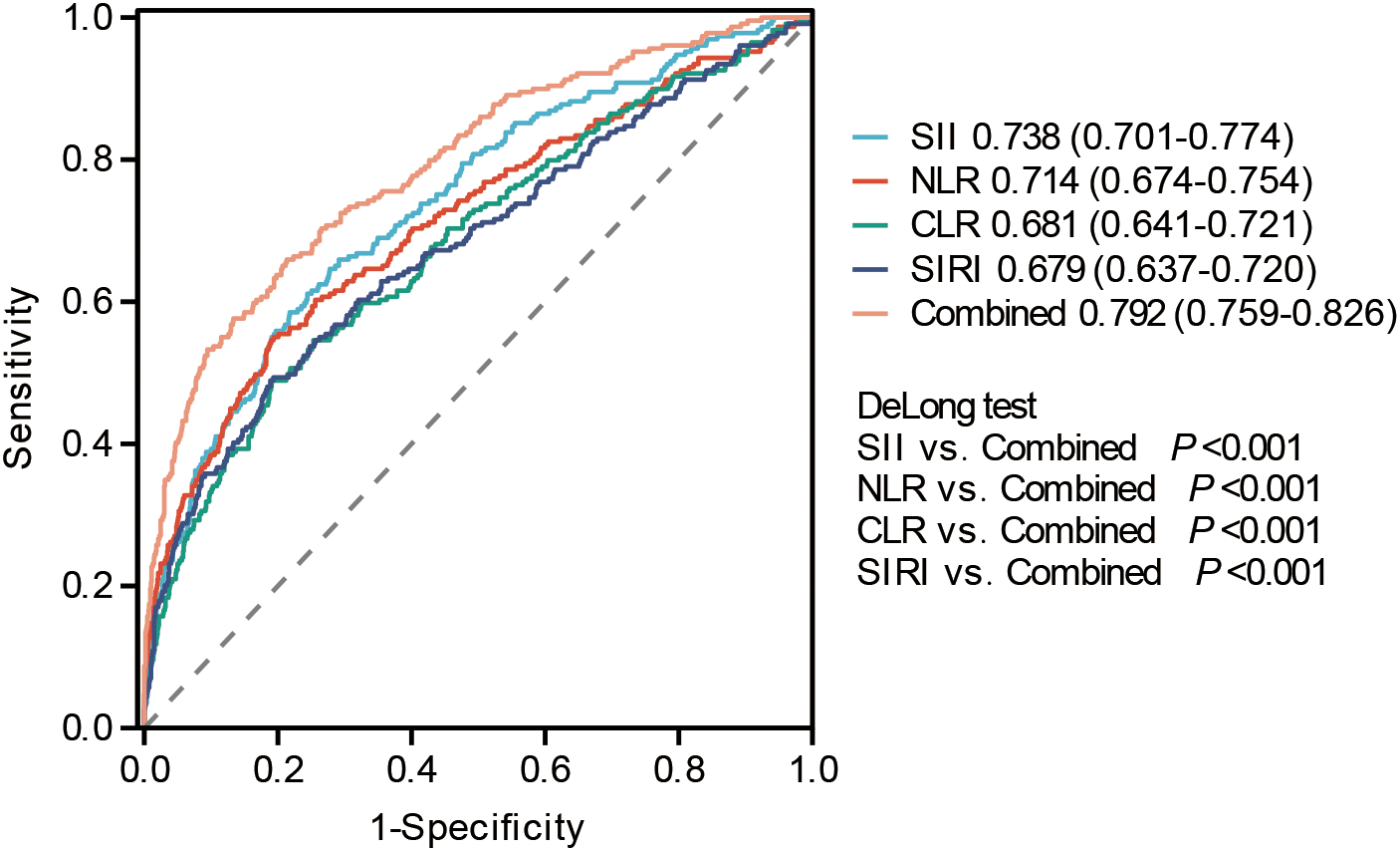
ROC curve evaluates the predictive performance of SII, NLR, CLR and SIRI combination the risk of POCs. SII, Systemic immune-inflammation index; NLR, Neutrophil-to-lymphocyte ratio; CLR, C-reactive protein-to-lymphocyte ratio; SIRI, Systemic inflammation response index.

**Figure 3:**
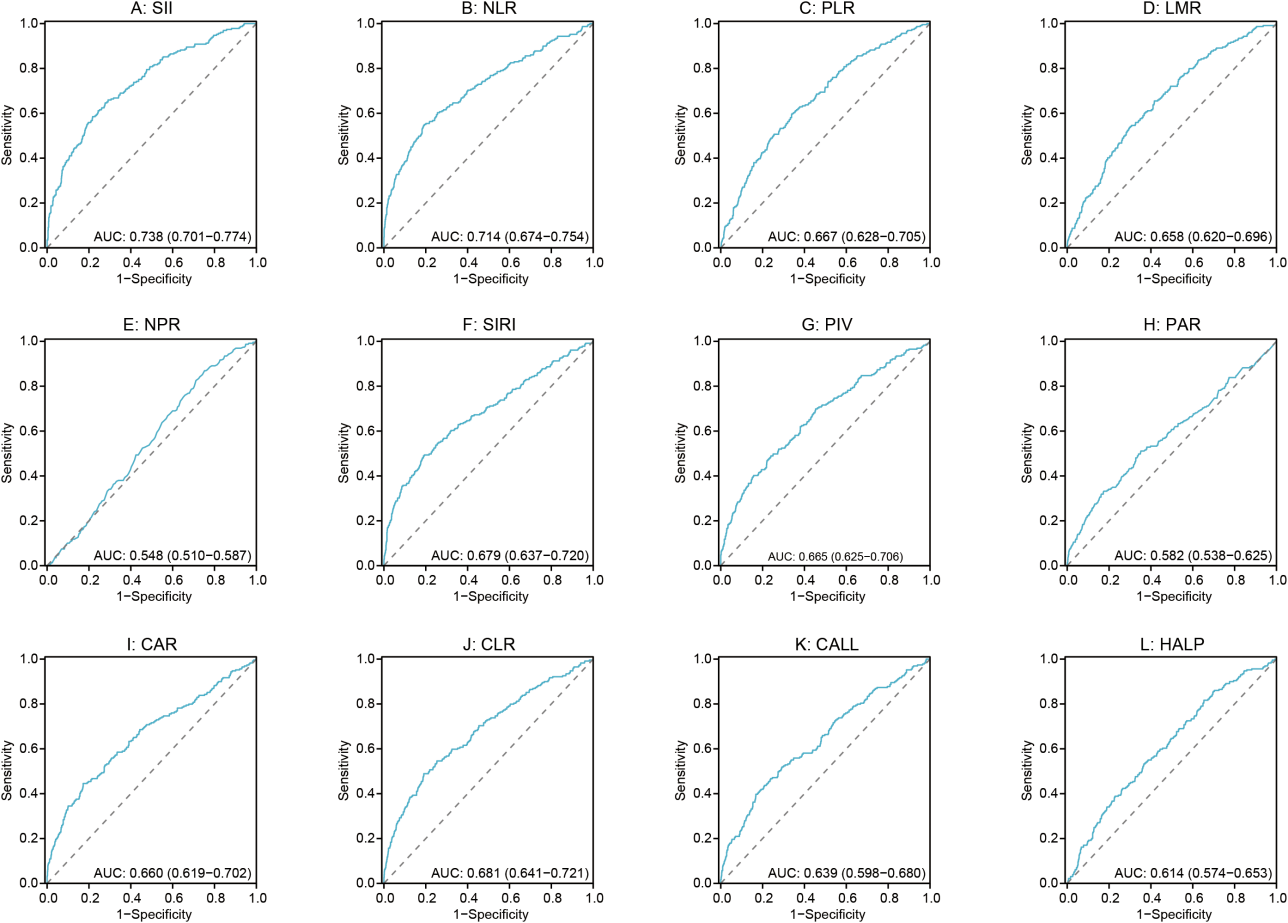
ROC curve evaluates the predictive performance of composite inflammatory indices and the risk of POCs. SII, Systemic immune-inflammation index; NLR, Neutrophil-to-lymphocyte ratio; PLR, Platelet-to-lymphocyte ratio; LMR, Lymphocyte-to-monocyte ratio; NPR, Neutrophil-to-platelet ratio; SIRI, Systemic inflammation response index; PIV, Pan-immune inflammation value; PAR, Platelet-to-albumin ratio; CAR, C-reactive protein-to-albumin ratio; CLR, C-reactive protein-to-lymphocyte ratio; CALLY, C-reactive protein-albumin-lymphocyte indices; HALP, Hemoglobin-albumin-lymphocyte-platelet.

The discriminative ability of each preoperative inflammatory index for POCs is shown in **Figure 2**. Among the 12 indices evaluated, four demonstrated higher AUC values: the SII (AUC: 0.738, 95% CI: 0.701-0.774), NLR (AUC: 0.714, 95% CI: 0.674-0.754), CLR (AUC: 0.681, 95% CI: 0.641-0.721), and SIRI (AUC: 0.679, 95% CI: 0.637-0.720). To enhance predictive performance, a combined model was constructed by integrating these four top-performing indices (SII, NLR, CLR, and SIRI). As presented in **Figure 3**, this combined model achieved a significantly superior discriminative ability, with an AUC of 0.792 (95% CI: 0.759-0.826). DeLong’s test for the comparison of ROC curves confirmed that the AUC of the combined model was statistically significantly higher than that of each constituent single index (all *P* < 0.05). The cut-off value, sensitivity, specificity, and Youden index for composite inflammatory index and the combined model are presented in **Table 5**.

**Table 5:** Cut-off value, sensitivity, specificity, and Youden index for preoperative composite inflammatory indices and the combined model.

Variable	Cut-off value	Sensitivity	Specificity	Youden index
Composite inflammatory indices				
SII	702.34	0.646	0.722	0.368
NLR	3.27	0.650	0.707	0.357
PLR	168.55	0.598	0.660	0.258
LMR	2.91	0.655	0.583	0.238
NPR	–	0.569	0.548	0.117
SIRI	1.72	0.689	0.611	0.300
PIV	425.18	0.589	0.660	0.249
PAR	6.83	0.511	0.648	0.159
CAR	0.41	0.645	0.628	0.273
CLR	9.26	0.589	0.709	0.298
CALLY	11.38	0.597	0.632	0.229
HALP	398.47	0.533	0.633	0.166
Combined				
SII+NLR+CLR+SIRI	–	0.659	0.787	0.446

SII, Systemic immune-inflammation index; NLR, Neutrophil-to-lymphocyte ratio; PLR, Platelet-to-lymphocyte ratio; LMR, Lymphocyte-to-monocyte ratio; NPR, Neutrophil-to-platelet ratio; SIRI, Systemic inflammation response index; PIV, Pan-immune inflammation value; PAR, Platelet-to-albumin ratio; CAR, C-reactive protein-to-albumin ratio; CLR, C-reactive protein-to-lymphocyte ratio; CALLY, C-reactive protein-albumin-lymphocyte indices; HALP, Hemoglobin-albumin-lymphocyte-platelet.

## 4 Discussion

The principal findings of this two-center retrospective study elucidate the substantial and independent prognostic value of readily available composite inflammatory indices for predicting POCs following curative-intent CRC surgery. Our comprehensive analysis of 12 distinct indices revealed a consistent and profound preoperative dysregulation of the immune-nutritional milieu in patients who subsequently developed complications. Notably, the SII, NLR, CLR, and SIRI emerged as the most robust standalone predictors, exhibiting strong, graded associations with complication risk even after rigorous adjustment for established clinical confounders. A model combining these four markers demonstrated superior predictive capacity, underscoring the complementary information captured by these indices and highlighting a promising multi-faceted approach to preoperative risk stratification.

Our findings provide robust, comparative evidence that extends the existing literature, which has predominantly focused on the prognostic role of indices like NLR and PLR in long-term oncological outcomes (19–21). The superior performance of the SII as a standalone predictor is mechanistically grounded. Its formula integrates three synergistic pathological pathways activated by surgical stress: neutrophilic inflammation, platelet-mediated pro-thrombotic shift, and lymphocytopenic immunosuppression (12, 22). This tripartite design captures a converging network of biological dysfunction more comprehensively than dual-parameter ratios. Specifically, neutrophilia drives tissue damage via protease and reactive oxygen species release, while thrombocytosis amplifies microthrombosis risk (23). Concurrent lymphocytopenia reflects attenuated adaptive immune responses, compromising host defense against pathogens and impairing wound healing coordination (24).

Equally insightful is the independent predictive value of the CLR. Unlike the more commonly studied CRP-to-albumin ratio, which contrasts inflammation with nutritional status, CLR directly opposes the magnitude of the humoral acute-phase response against the capacity of cellular immunity (25). A high CLR likely denotes a state where inflammatory signals overwhelm lymphocyte-mediated repair and defense mechanisms (26, 27). This imbalance may facilitate a transition from contained, reparative inflammation to a persistent and damaging systemic response, predisposing to septic complications and poor tissue anastomosis healing (28).

The compelling clinical-pathophysiological correlation observed, marked by elevated pro-inflammatory indices and depressed indices of immune-nutritional reserve in patients with complications, strongly reinforces the central hypothesis. It posits that an excessive or imbalanced preoperative systemic inflammatory response is a critical, modifiable driver of morbidity (29). Patients entering surgery with this heightened inflammatory set-point exhibit a diminished capacity for regulated healing. Instead, they are predisposed to a maladaptive cascade wherein dysregulated inflammation impairs tissue repair, increases vascular permeability, promotes a pro-thrombotic state, and suppresses adaptive immunity (30). The exceptional predictive performance of SII and NLR derives from their quantitative integration of the very drivers of inflammation and the markers of immune compromise that underpin this pathophysiology (31).

A principal implication of our study is the demonstrably superior accuracy achieved by the combined model incorporating SII, NLR, CLR, and SIRI. This result reinforces the understanding that POCs are the product of multifaceted, interacting physiological disturbances (32, 33). Each individual index captures a distinct, though overlapping, facet of this complex landscape. Their integration yields a more stable and holistic risk profile, offsetting the variability inherent in single biomarkers. The model’s discriminative power, exceeding that of conventional clinical factors alone, supports the potential clinical integration of such biomarker panels into preoperative risk assessment (34, 35). This approach could facilitate earlier identification of high-risk patients, permitting targeted prehabilitation strategies. Such strategies may include nutritional optimization, physical pre-conditioning, and even pharmacological modulation of inflammation, followed by tailored postoperative monitoring protocols. This advances the paradigm of personalized perioperative medicine.

This study has several limitations. First, the retrospective design may introduce selection bias and residual confounding, despite enabling efficient analysis of a large dual-center cohort. Although we adjusted for key confounders, variables such as American Society of Anesthesiologists (ASA) score, intraoperative blood loss, and tumor size were not consistently captured across both centers. Future prospective, multicenter studies with standardized data collection are needed to validate our findings and account for these unmeasured confounders. Second, reliance on a single preoperative blood measurement, though clinically practical, precludes assessment of perioperative dynamics that could offer additional prognostic insight. Serial perioperative measurements in future studies may clarify the temporal evolution of these indices and their relationship with postoperative recovery. Third, our findings may not generalize to patients receiving neoadjuvant therapy, as the cohort was restricted to those undergoing elective minimally invasive surgery without such treatment. Further investigations in patient populations receiving neoadjuvant therapy are warranted to assess the generalizability of our results. Finally, the identified indices and proposed cut-off values require external validation in prospective cohorts to confirm clinical utility and establish generalizable, actionable thresholds. Future studies should consider integrating these indices into multimodal predictive models and evaluating their utility in biomarker-guided prehabilitation strategies.

## 5 Conclusion

In conclusion, this study establishes that preoperative composite inflammatory indices, notably SII, NLR, CLR, and SIRI, serve as robust and independent predictors of complications following CRC surgery. These indices objectively quantify a patient’s baseline inflammatory and immune status. Crucially, a model combining these markers demonstrated superior predictive performance, indicating that a multi-parametric biomarker panel outperforms reliance on any single index. Our work clarifies the predictive hierarchy among numerous indices and validates newer markers such as CLR. Future prospective research is needed to define clinical cut-offs, integrate these tools into decision-support systems, and evaluate whether biomarker-guided risk stratification can meaningfully reduce postoperative morbidity and improve patient outcomes.

## Data Availability

The original contributions presented in the study are included in the article/supplementary material. Further inquiries can be directed to the corresponding authors.
